# Multimodal Attention Dynamic Fusion Network for Facial Micro-Expression Recognition

**DOI:** 10.3390/e25091246

**Published:** 2023-08-22

**Authors:** Hongling Yang, Lun Xie, Hang Pan, Chiqin Li, Zhiliang Wang, Jialiang Zhong

**Affiliations:** 1 Department of Computer Science, Changzhi University, Changzhi 046011, China; honglingyang0@gmail.com; 2 School of Computer and Communication Engineering, University of Science and Technology Beijing, Beijing 100083, China; emotiongroupxie@163.com (L.X.); lcqin111@163.com (C.L.); wzl@ustb.edu.cn (Z.W.); 3 School of Mathematics and Computer Sciences, Nanchang University, Nanchang 330031, China; 416100210107@email.ncu.edu.cn

**Keywords:** micro-expression recognition, learnable class token, dynamic fusion

## Abstract

The emotional changes in facial micro-expressions are combinations of action units. The researchers have revealed that action units can be used as additional auxiliary data to improve facial micro-expression recognition. Most of the researchers attempt to fuse image features and action unit information. However, these works ignore the impact of action units on the facial image feature extraction process. Therefore, this paper proposes a local detail feature enhancement model based on a multimodal dynamic attention fusion network (MADFN) method for micro-expression recognition. This method uses a masked autoencoder based on learnable class tokens to remove local areas with low emotional expression ability in micro-expression images. Then, we utilize the action unit dynamic fusion module to fuse action unit representation to improve the potential representation ability of image features. The state-of-the-art performance of our proposed model is evaluated and verified on SMIC, CASME II, SAMM, and their combined 3DB-Combined datasets. The experimental results demonstrated that the proposed model achieved competitive performance with accuracy rates of 81.71%, 82.11%, and 77.21% on SMIC, CASME II, and SAMM datasets, respectively, that show the MADFN model can help to improve the discrimination of facial image emotional features.

## 1. Introduction

Facial micro-expressions (hereinafter referred to as micro-expressions) are short-duration and low-intensity facial muscle movements. Since it usually occurs when hiding emotion in the heart and can reflect genuine emotions and motivations [1]. If people are not professionally trained, it is impossible to hide the appearance of micro-expressions [2]. Researchers found that micro-expressions are often present in lie detection scenarios. Thus, it has major implications when it comes to high-risk situations, including criminal investigation, social interactions, national security, and business negotiations [3].

The researchers have shown that facial emotional changes are a combination of some action units (AUs), which can be used as additional auxiliary information to improve the performance of facial micro-expressions recognition [4]. Xie et al. [5] combined AU detection and micro-expression recognition and proposed an AU-assisted Graph Attention Convolutional Network. The model predicts micro-expression categories by learning AUs node features in the graph convolutional network learning module. Lei et al. [6] proposed a graph convolutional network based on AU, which enhanced the feature representation of nodes and graph edges extracted by the graph convolutional network by fusing AU features. Zhao et al. [7] proposed a Spatio-Temporal AU Graph Convolution Network, which inputs local image regions of AUs to a three-dimensional convolutional model to obtain AU features. They tried to utilize graph convolutional networks to focus on the dependencies between different local regions to improve the performance of micro-expressions recognition.

**Figure 1:**
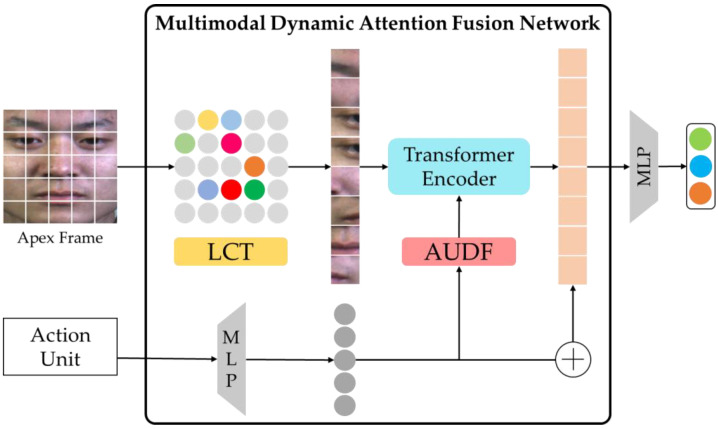
The framework of the MADFN method. The apex frame of the micro-expression video clip is input to an LCT module to remove local areas with low emotional expression. Then, the AUDF module adds to the vision transformer encoder to fuse action unit representation to improve the potential representation ability of image features. Finally, the local image features with high attention weight are fused action unit representations for micro-expression recognition.

Although these methods employ action units to enhance image features, they do not consider the impact of action units on the image feature extraction process. The studies have shown that the Vision Transformer (ViT) [8] model can achieve success in tasks such as image recognition [9], object detection [10], image segmentation [11], and generation [12] by focusing on image local information. The ViT model structure can introduce auxiliary information into the image encoder model to dynamically enhance features. Therefore, this paper proposes a local detail feature enhancement method based on a multimodal dynamic attention fusion network (MADFN) model for micro-expression recognition. This model of the local detail feature enhancement method is shown in Figure 1.

In this model, a learnable class token (LCT) is used to remove local areas with low emotional expression ability in micro-expression images. To enhance the discrimination of emotional features, the action unit representation is added to the extraction process of extracting the potential emotional features of the image, and the action unit dynamic fusion (AUDF) module is used to fuse the action unit representation with the local features of the image sub-blocks with high weight for micro-expression recognition. We evaluated the MADFN model on three datasets: Spontaneous Micro-expression Corpus (SMIC) [13], Chinese Academy of Sciences Micro-Expression II (CASME II) [14], Spontaneous Actions, and Micro-Movements (SAMM) [15], and their combined dataset (3DB-combined) [16].

In general, this paper attempts to propose a MADFN model for solving the local detail feature enhancement problem. The main contributions of this paper are summarized as follows:

1. The masked autoencoder based on the learnable class token is proposed to remove small contributing local image sub-blocks for micro-expression recognition.

2. The influence of action units on facial micro-expression recognition is analyzed, and we are the first to add action unit representations to the feature extraction process of micro-expression images.

The remainder of this paper is organized as follows: In Section 2, a brief review of the related research on micro-expression recognition. Section 3 provides a complete introduction to the proposed model. Section 4 shows the datasets, details, and results of the experiment. Finally, Section 5 presents the conclusions of this research method.

## 2. Related Work

The facial micro-expression recognition methods are generally divided into two types. The first type of method extracts global features from the whole image for micro-expressions recognition; meanwhile, the second type of method locates the local regions where micro-expressions occur and then extracts local features for micro-expression recognition.

### 2.1. Global Features for Micro-Expression Recognition

Several earlier studies [17,18,19,20,21] that make hand-crafted features adequately represent the micro-expression changes on facial micro-expression recognition used a rule-based block division approach to extract features from each block to be stitched into a compact feature vector for micro-expression recognition to make hand-crafted features best represent micro-expression changes [22,23]. This methodology was first used for micro-expression recognition by Pfister et al. [17]. The micro-expression video images were uniformly separated into 4 × 4, 5 × 5, and 6 × 6 blocks evenly from the three planes of XY, XT, and YT. To recognize the micro-expressions, the Local Binary Pattern (LBP) features of these blocks are extracted and combined into a feature vector. Wang et al. developed the Local Binary Pattern with Six Intersections Point (LBP-SIP) to reduce the information redundancy of the LBP-TOP feature and, thus, the time-space complexity. Spatio-Temporal Local Binary Pattern with Integral Projection (STLBP-IP) was proposed by Huang et al. to enhance the properties of LBP-TOP through integrated projection. By using Sparsity-Promoting Dynamic Mode Decomposition (DMDSP) to remove neutral expressions from micro-expression videos, Le Ngo et al. managed to achieve a high recognition rate. To overcome the sparsity problem of the LBP features, Huang et al. [20] utilized the same procedure to divide the micro-expression video images and thereafter extract the Spatiotemporal Completed Local Quantized Patterns (STCLQP) features of each region block. Wang et al. [24] explored the rule-based block division method in different color spaces to verify the influence of the color feature spaces on micro-expression recognition.

Although hand-crafted feature methods may give excellent micro-expression recognition results, they can ignore additional information in the original image data. With the development of deep learning, researchers consider applying it in micro-expression recognition to extract subtle changes in the features of micro-expression [25,26,27]. Kim et al. [28] employed a Recurrent Neural Network to extract the temporal features of the micro-expression video images for micro-expression recognition while using the Convolutional Neural Networks (CNN) architecture to capture the spatial information from different temporal stages (onset, apex, and offset frame). Liong et al. [29] developed an optical flow feature from the apex frame (OFF-apex) framework, which utilizes the optical flow feature map of the micro-expression apex frame as the input of the CNN to enhance the optical flow features and improve the recognition rate of micro-expressions. Micro-expression recognition using deep learning methods is the favorite choice of researchers with excellent results in the 2019 Facial Micro-Expression Grand Challenge (MEGC 2019) [30,31,32,33].

### 2.2. Local Features for Micro-Expression Recognition

Although, the global image feature extraction method can improve effectiveness in micro-expression recognition. However, this may neglect the influence of local information and also brings the problem of information redundancy. Therefore, researchers first locate regions where micro-expressions occur and then extract local features of these regions for micro-expression recognition. The initial work on local features moved away from a rule-based block division approach and toward a rule-based facial ROI features extraction [34,35]. Wang et al. [36] used the Facial Action Coding System (FACS) to distinguish 16 ROIs and obtained the Local Spatiotemporal Directional features of these regions through Robust Principal Component Analysis (RPCA) for micro-expression recognition. Liu et al. [37] proposed a Main Directional Mean Optical (MDMO) flow feature. To reduce the impact of noise caused by head movement in micro-expression recognition, this method employs the robust optical flow method to extract features from 36 ROIs divided by Action Units (AU) in micro-expression video images. Xu et al. [38] suggested a micro-expression recognition method based on the Facial Dynamics Map (FDM), which locates ROIs based on facial emotion in a micro-expression video sequence and extracts features from these regions for micro-expression recognition. Happy et al. [39] employed the FACS to locate 36 facial ROIs and applied the Fuzzy Histogram of Optical Flow Orientation (FHOFO) method to extract the subtle changes features in these regions of these regions for micro-expression recognition. Liong et al. [40] presented a Bi-Weighted Oriented Optical Flow (BI-WOOF) feature descriptor, which uses two schemes to perform a weighted average of the global and local Histogram of Oriented Optical Flow (HOOF) features. Each ROI is weighted using the magnitude component and multiplied by the average optical variation of each ROI amplitude in the local feature extraction. The final histogram features are weighted from the overall HOOF features for micro-expression recognition.

Although the rule-based ROIs location method can help improve the recognition accuracy of the micro-expressions, it may not obtain the best results. Therefore, researchers use deep learning or attention mechanisms to obtain local features to recognize micro-expressions. Chen et al. [41] introduced a three-dimensional spatiotemporal convolutional neural network with a Convolutional Block Attention Module (CBAM) for micro-expression recognition, which included a visual attention mechanism. While this method focuses on the importance of the features of interest, it ignores the subtle feature of the local regions. Li et al. [42] presented an LGCcon learning module, which combines local and global information to discover local regions of key emotional information while suppressing the detrimental impact of irrelevant facial regions on micro-expression recognition. Wang et al. [43] presented a Residual Network with Micro-Attention (RNMA) model to locate the facial ROIs holding distinct AU to address the influence of micro-expression changes in local regions. Xia et al. [44] proposed a recurrent convolutional network (RCN) to explore the effects of shallow architecture and low-resolution input data on micro-expression recognition using an attention model for focusing on local facial regions.

## 3. Methodology

### 3.1. Multimodal Dynamic Attention Fusion Network

The multimodal dynamic attention fusion network consists of two inputs micro-expression image and AU embedding. First, the micro-expression image is divided into regular non-overlapping sub-blocks. The mask operation is performed on the image sub-blocks that contribute less to micro-expression recognition through the learnable class token module. The image sub-blocks with high attention weights are input into the action unit dynamic fusion module through normalization and multi-head self-attention (MSA) operations and fused with AU embedding to improve the distinguishability of high-dimensional local feature representation of micro-expression images. Finally, the final micro-expression prediction is performed by fusing AU embedding and enhanced image local representation.

**Figure 2:**
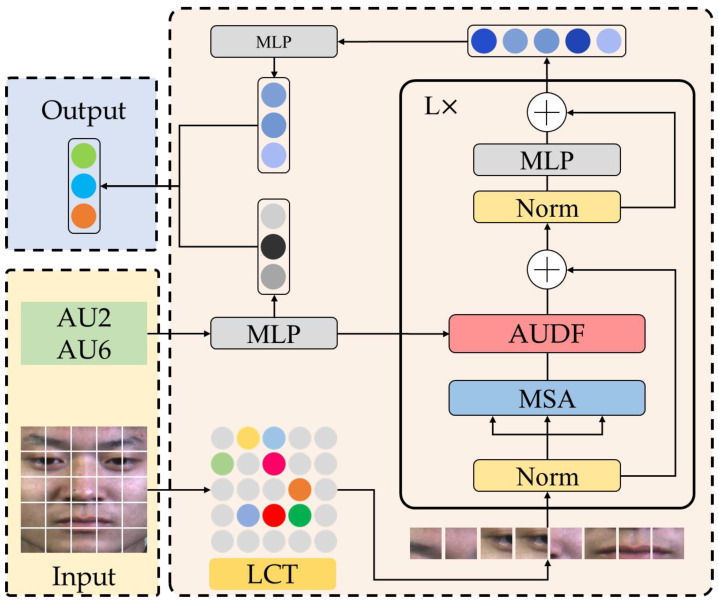
The framework structure of the multimodal dynamic attention fusion network.

Different from the fusion methods of feature connection, addition, or multiplication, this paper embeds the action unit dynamic fusion module into the transformer encoder model and uses AU embedding to enhance the local feature representation of micro-expression image, thereby increasing the discrimination of image features to improve micro-expression recognition performance. The framework structure of the multimodal dynamic attention fusion network is shown in Figure 2.

### 3.2. Image Autoencoders Based on Learnable Class Token

The problem of small datasets for micro-expressions severely limits model fitting, while the micro-expression image needs to be divided into regular non-overlapping image sub-blocks in a multimodal dynamic attention fusion network model. If these image sub-blocks are directly input to the visual transformer, it will lead to information redundancy. The low intensity of micro-expression movement results in slight differences between images of a subject in different categories but huge differences between images of different subjects within the same category. Therefore, micro-expression recognition can be regarded as a fine-grained image classification problem, and more attention should be paid to the distinguishability of local image features.

For the local perception of fine-grained image classification, He et al. [45] proposed a Masked Autoencoder (MAE) model, which adopts a random sampling (RS) module to mask a large number of image sub-blocks to reduce redundancy. Compared with block-wise sampling (BS) and grid-wise sampling (GS) modules, random sampling can construct efficient feature representation through highly sparse image sub-blocks. However, the uncertainty of random sampling may remove some image sub-blocks with high representational power.

**Figure 3:**
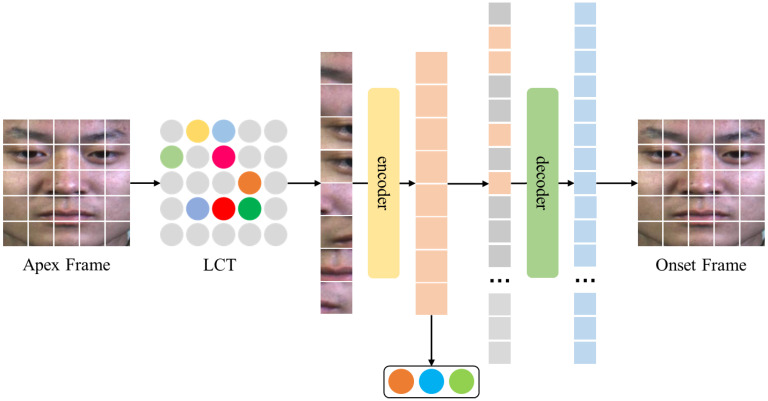
The image autoencoder structure is based on a learnable class token.

Therefore, this section proposed an image autoencoder pre-training model based on a learnable class token. The model structure is shown in Figure 3. The model utilizes a learning sampling (LS) module to remove local image sub-blocks that contribute little to micro-expression recognition, reducing the complexity of the pre-training model and improving model performance while focusing on the emotional feature representation of local areas.

The image autoencoders-based learnable class tokens are an end-to-end pre-training model. The model iteration is divided into two parts. Firstly, all the images of the micro-expression video samples are input to the autoencoder for training to obtain the high-dimensional representations of the facial image.

Specifically, the image is divided into regular non-overlapping image patches $x_{p}$. These image blocks are masked by the LCT module, and the high-weight image sub-blocks are input to the encoder network. The encoder uses the image path of a multimodal dynamic attention fusion network to extract the representations of local sub-blocks. Then, these representations and the learnable class token are reconstructed according to the original position and input to the decoder network to restore the original image. In the second iteration, the apex frame is input to the autoencoder, and the output onset frame extracts the emotional representation in the apex frame for micro-expression recognition.

The LCT module is a fully connected layer model in which the input is a feature vector with the same length as the image sub-blocks $x_{p}$, and the output is sorted to remove those corresponding low-weight images. This module specifically expressed as

(1) $$x_{s}=x_{p}*{\left[{w_{k},\cdots ,w}_{i},\cdots ,w_{p}\right]}^{T},$$

(2) $$w_{i}=\left\{\begin{array}{c} 1,\;if\;w_{lmt\_i}\geq \theta \\ 0,\;if\;w_{lmt\_i}<\theta \end{array}\right.,$$

(3) $$\theta =\mu *rank\left(w_{lmt}\right),$$

where, $x_{s}$ is the image sub-block sampled by the LCT module, $w_{lmt}$ is the parameter of the LCT module, $w_{i}$ is the binary mask token projection corresponding to each image sub-block, θ is the division of the mask weight threshold, and μ is the proportion of all image sub-blocks masked by the LCT model.

In the model pre-training process, the parameters are updated through two different loss functions, which are expressed as follows:

(4) $$l_{i}=\frac{1}{n}\sum_{i=1}^{n}{\left(x_{i}-{\tilde{x}}_{i}\right)}^{2},$$

(5) $$l_{o}=\frac{1}{n}\sum_{i=1}^{n}{\left(x_{o}-{\tilde{x}}_{o}\right)}^{2},$$

where, $l_{i}$ is the loss function of image autoencoder, $x_{i}$ is the *i*-th frame image in the micro-expression video sample, $l_{o}$ is the loss function of apex frame to onset frame mapping, $x_{o}$ is the onset frame, ${\tilde{x}}_{i}$ and ${\tilde{x}}_{o}$ are corresponding generated face images.

Different from the random sampling of image sub-blocks in the MAE model, this paper removes low-weight image sub-blocks through a learnable class token module. The learned mask sub-blocks are rearranged in the order in which the sub-blocks were removed. Finally, masked subblocks with low weights are again selected for deletion. This cycle repeats until the best high-weight local region is selected for micro-expression recognition. This learnable method reduces information redundancy to a large extent by deleting a large number of image sub-blocks.

### 3.3. Vision Transformer Model Based on Action Unit Dynamic Fusion

**Figure 4:**
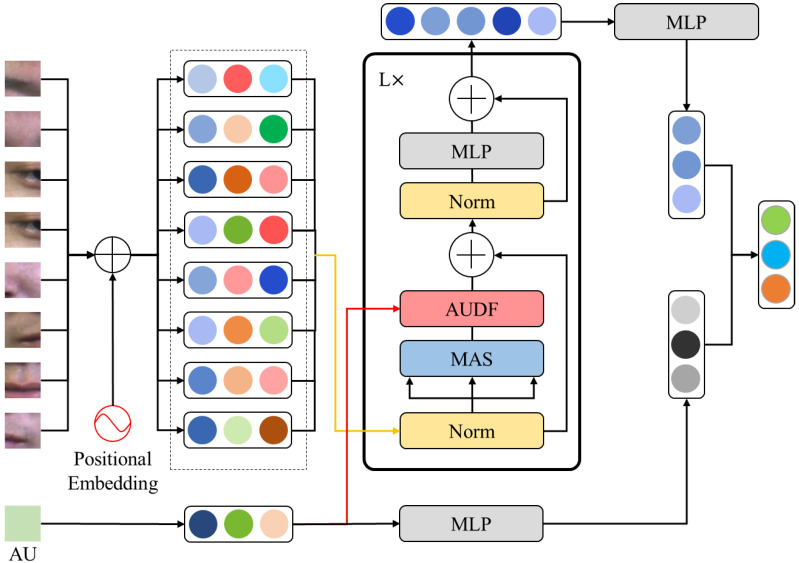
The vision transformer model is based on the action unit dynamic fusion structure.

Due to the low intensity of micro-expression facial motion, it is difficult to obtain highly discriminative local representations, which affects the performance of facial micro-expression recognition. The ViT model has been widely used in computer vision [46]. The studies have shown that in image classification tasks, the ViT model can improve recognition performance by focusing on the attention weights of image sub-blocks. However, due to the complexity of the network structure, the ViT model usually requires large-scale data for model training. Therefore, we first utilize a large number of mask operations on the image through the LCT module to reduce the complexity of the model. Then, the representations of the reserved image sub-blocks are input into the vision transformer model based on action unit dynamic fusion to fuse the facial AU embedding to recognize the emotional state of the face. The vision transformer model based on the action unit dynamic fusion structure is shown in Figure 4.

**Figure 5:**
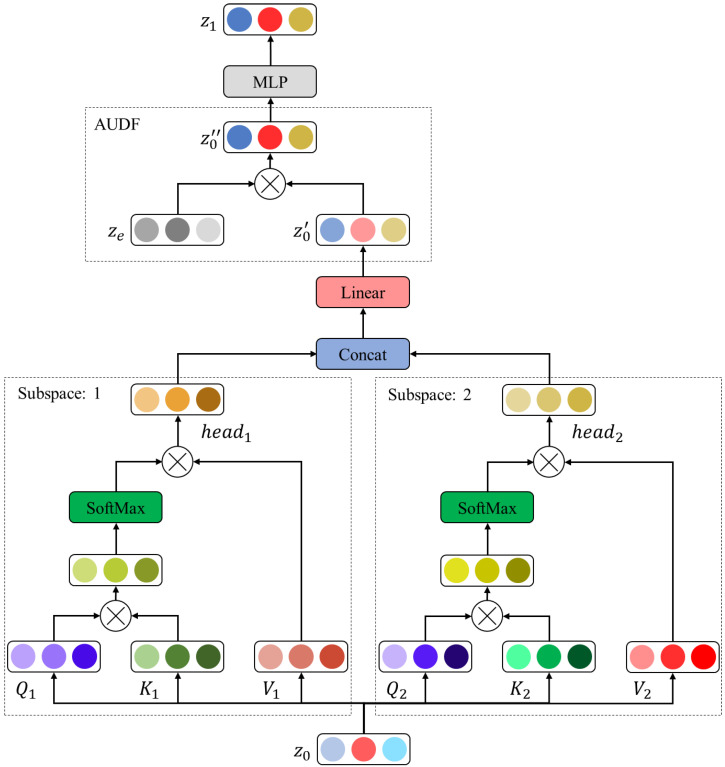
The single-layer MSA, AUDF, and MLP model structures.

The improved ViT encoder includes L-layer MSA, AUDF, and MLP modules. The single-layer MSA, AUDF, and MLP model structures are shown in Figure 5.

Firstly, the image representations $z_{0}$ of the remaining image, sub-blocks are normalized and input to the MSA model to calculate the attention weight of each image sub-block. For each subspace, define three feature matrices $W_{Q,i}$, $W_{K,i}$ and $W_{V,i}$ to linearly map image sub-blocks and obtain matrix query $Q_{i}$, key $K_{i}$, and value $V_{i}$ in the MSA module. Then, $Q_{i}$ and $K_{i}$ perform the dot product operation to obtain the attention probability distribution of each image sub-block through SoftMax, and then multiply it with the value matrix to obtain the attention weight of the image sub-block. Finally, the weights of each subspace in MSA are concatenated and multiplied to obtain the final feature output ${z_{0}}^{\prime }$.

(6) $${z_{l}}^{\prime }=MSA\left(LN\left(z_{l-1}\right)\right)+z_{l-1},\;l=1,\ldots ,L,$$

(7) $$MSA=LN\left(Concat\left({head}_{1},{head}_{2},\cdots ,{head}_{k}\right)\right),$$

(8) $${head}_{i}=Softmax\left(\frac{Q_{i}K_{i}^{T}}{\sqrt{d_{k}}}\right)V_{i}$$

(9) $$Q_{i}=z_{l-1}W_{Q,i},\;i=1,\ldots ,k$$

(10) $$K_{i}=z_{l-1}W_{K,i},\;i=1,\ldots ,k$$

(11) $$V_{i}=z_{l-1}W_{V,i},\;i=1,\ldots ,k$$

The AU embedding has been proven to help extract more effective feature representations in micro-expression recognition, but how to dynamically add AU information to the process of feature extraction is still blank in the current research field. Inspired by dynamic filters [47,48,49,50], this paper proposes an action unit dynamic fusion module to add AU embedding to a vision transformer encoder model for enhancing the discriminability of micro-expression image representations.

In the basic AUDF module, the AU-encoded features are first replicated with the same number of image sub-blocks and then multiplied with the output of MSA. The AUDF module utilizes dynamic multiplication to fuse AU embedding into the local feature extraction process to increase the discrimination of facial emotional representations. The calculation method is as follows:

(12) $${z_{l}}^{\prime \prime }=AUDF\left(LN\left({z_{l}}^{\prime }\right)\right)+{z_{l}}^{\prime },\;l\in 1,\ldots ,L,$$

(13) $$AUDF=Reshape\left(LN\left(z_{e}\right)\right)\times {z_{l}}^{\prime },$$

where, ${z_{l}}^{\prime }$ is the output image local attention representations weight of the MSA model, ${z_{l}}^{\prime \prime }$ is the output of the AUDF module, $z_{e}$ is the additional facial AU embedding, Reshape() is to transform the one-dimensional feature is a two-dimensional matrix, and LN() represents a fully connected layer.

However, facial representation and AU embedding are mutually complementary and interdependent in micro-expression recognition. Therefore, to further enhance the enhancement effect of AU embedding on facial representations, this paper introduces an AUDF enhancement module, AUDF-E.

First, the attention weight output by MSA is down-sampled and mapped to a one-dimensional feature $z_{i}$. Then, perform a splicing operation with the AU embedding $z_{e}$ and then linearly change it to the same dimension feature as each local image sub-block. Finally, the features copied and concatenated are multiplied by the output of MSA with the same number of image sub-blocks. AUDF-E is expressed as follows:

(14) $$AUDF-E=Reshape\left(LN\left(\left[z_{i},z_{e}\right]\right)\right)\times {z_{l}}^{\prime },$$

(15) $$z_{i}=Pool\left(z_{l-1}\right),$$

The AUDF module is used to dynamically enhance the local image representations, and the facial emotion representations are obtained through the MLP module by residual connection and normalized. Finally, the output of the vision transformer encoder is fused with the AU embedding to obtain the final classification result.

(16) $${z_{l}}^{\prime \prime }=LayerNorm\left(z_{l-1}+{z_{l}}^{\prime \prime }\right),$$

(17) $$z_{l}=MLP\left(LN\left({z_{l}}^{\prime \prime }\right)\right)+{z_{l}}^{\prime \prime },$$

(18) $$p=Softmax\left(MLP\left(z_{L}^{0}\right)+MLP\left(z_{e}\right)\right)$$

where p is the micro-expression prediction probability of the MADFN model, $z_{L}^{0}$ is the category label output. Finally, the class token $z_{L}^{0}$ is replaced with the parameters corresponding to the micro-expression of LCT, and the comparison sub-block with high attention weight is used for the next iteration. During the MADFN model training process, the focal loss is used to reduce the impact of category imbalance.

## 4. Results and Analysis

In this section, the analysis and comparison of experimental results, ablation experimental analysis, and visualization analysis will be introduced in detail. The proposed MADFN model was verified experimentally on three public facial micro-expression datasets SMIC, CASME II, SAMM, and their combination 3DB-combined.

### 4.1. Quantitative Analysis

#### 4.1.1. SMIC

Table 1 shows the comparison results of the MADFN model on the SMIC dataset and two types of baseline methods for three classifications. The accuracy of the MADFN model is 6.07% and 11.2% higher than the best KTGSL in the global feature method and the best model SMDMO based on local features, respectively. The F1-Score of our model is higher than the best TSCNN model in the global feature method, and the best model SMDMO based on local features is 0.0966 and 0.1161, respectively. The effectiveness of the MADFN model is demonstrated by comparing it with two classes of baseline methods.

#### 4.1.2. CAMSE II

Table 2 shows the comparison results of the MADFN model on the CAMSE II dataset and two types of baseline methods for three classifications. Compared with local feature methods, the MADFN model outperforms existing baseline methods. Although the MERSiamC3D model with global features is 0.0205 higher than the F1-Score performance of the MADFN, the MERSiamC3D model uses key frame images in video sequences for recognition, and the model structure is more complex. Compared with the optimal TSGACN method in local features, the strategy of fusing facial key points and optical flow features, the MADFN enhances facial local features through AU embedding, and the accuracy and F1-Score performance indicators of the model are higher than TSGACN 0.41% and 0.6641. At the same time, the experimental results found that although TSGACN can achieve higher recognition accuracy, its F1-Score performance is slightly lower, which shows that they did not consider the influence of sample imbalance in the CAMSE II, and similar results emerged in the SAMM dataset.

#### 4.1.3. SAMM

Table 3 shows the comparison results of the MADFN model on the SAMM dataset and two types of baseline methods for three classifications. Compared with the global feature method, MADFN has achieved the best experimental results. However, in comparison with the method of local features, MADFN is still much different from the TSGACN model. The TSGACN has achieved excellent performance on the SAMM dataset with its unique model, but they are more focused on improving model performance. The recognition performance of the MADFN model is slightly inferior to that of TSGACN, but the MADFN model proposed in this paper pays more attention to the generalization ability, the processing of unbalanced data, and the complexity of the model. The experimental results show that MADFN can improve the classification accuracy even by fusing the overall and local features and alleviating the problem of micro-expression sample imbalance.

#### 4.1.4. MEGC2019

Table 4 shows the comparison results of the MADFN model on the MEGC2019 dataset and two types of baseline methods for three classifications. On the three subsets of SMIC, CASME II, and SAMM datasets, the MADFN proposed in this paper achieves the SOTA recognition results. At the same time, in the comparison experiment of the combined dataset 3DB-Combined, the MADFN model also achieved competitive performance. Compared with the optimal PLAN method in local features, MADFN also improves the UF1 and UAR indicators by 0.012 and 0.0024, respectively, which also proves the effectiveness of the model.

### 4.2. Ablation Experiment Analysis

A detailed analysis is carried out in the ablation experiments to evaluate the effectiveness of the local feature extraction of the MADFN. This section conducts ablation experiments in three aspects of the basic model, mask sampling strategy and fusion strategy in the SMIC, CASME II, and SAMM.

#### 4.2.1. Basic Model

This section first compares the influence of three different backbone networks of ViT Base (ViT-B/16), ViT Large (ViT-L/16), and ViT Huge (ViT-H/14) on micro-expression recognition. The backbone model network parameters are shown in Table 5.

It was found that in SMIC and SAMM, although the performance of ViT-H was higher than that of ViT-B structure, it was slightly inferior to that of ViT-L. In CASME II, ViT-H achieved the best results. The reason for this result is that the size of the micro-expression data set does not support training on a large-scale dataset but in a smaller-scale data set. Therefore, in the follow-up experiment process, this paper uses ViT-L as the backbone network for model training, and the experimental results are shown in Table 6.

#### 4.2.2. Mask Sampling

Based on determining the backbone network, the impact of different mask sampling strategies on the performance of the DViT model is further compared. Specifically, the impact of the four mask sampling strategies of BS, GS, RS, and LS is mainly compared. Among them, block sampling is to randomly mask out large image blocks, grid sampling refers to masking out three of every four small image blocks, and random sampling is large-scale masking out of small image blocks; a different sampling strategy is shown in the figure.

The experimental results are shown in Table 7. In the three comparison data sets, random sampling and learning sampling are much higher than average block sampling and grid sampling. Compared with the random adoption strategy, the learning sampling strategy can improve the accuracy of recognition. This is because mask sampling through learning can effectively avoid the uncertainty brought about by random mask sampling so that a highly differentiated local region of interest can be obtained through an accurate mask strategy, thereby extracting more robust local features to improve the performance of micro-expression recognition.

#### 4.2.3. Fusion Strategy

In the ablation experiment of AU-encoded feature enhancement, the effect of different fusion strategies on micro-expression recognition is mainly compared. First, the unimodal image data is fed into the ViT model with learned masks as a baseline comparison. Secondly, the local image features and AU embedding extracted by the ViT model are concatenated (ViT-C-AU), sum (ViT-S-AU), and multiplied (ViT-M-AU) for fusion. Finally, facial features are enhanced by fusing AU via the AUDF module.

The experimental results are shown in Table 8. Although the multi-modal feature concatenated, addition, and multiplied can improve the performance of micro-expression recognition, it cannot improve the discrimination of facial emotional features through AU embedding. The AUDF module proposed in this paper uses dynamic mapping to add AU embedding to the extraction process of facial emotional features and enhances emotional features. The proposed AUDF-E can achieve better experimental results by fusing facial image features and AU embedding.

### 4.3. Parameters

We have compared the total number of parameters, training, and testing time of the proposed model compared with existing models in the SMIC dataset using PyTorch on the GeForce RTX A6000 platform. The total parameters and time are shown in Table 9.

### 4.4. Visualization Analysis

**Figure 6:**
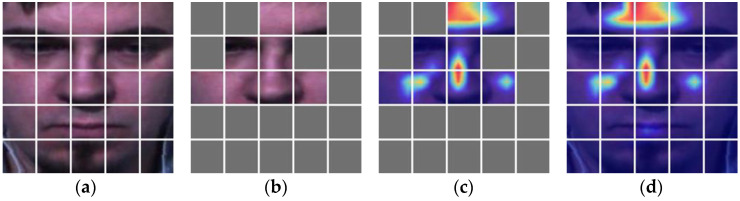
The correspondence between learnable class token and Grad-CAM in SMIC. (**a**) is a subblock of the original image; (**b**) is an image subblock that can learnable class token masks; (**c**) is the learnable class token mask image subblock corresponding to Grad-CAM; (**d**) is a Grad CAM image.

**Figure 7:**
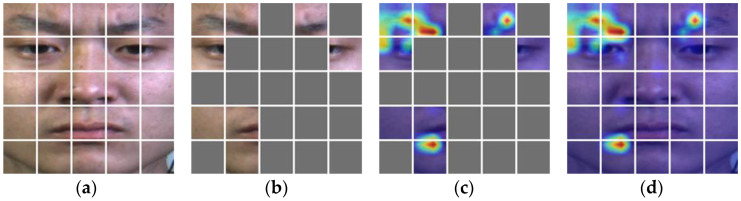
The correspondence between learnable class token and Grad-CAM in CASME II. (**a**) is a subblock of the original image; (**b**) is an image subblock that can learnable class token masks; (**c**) is the learnable class token mask image subblock corresponding to Grad-CAM; (**d**) is a Grad CAM image.

**Figure 8:**
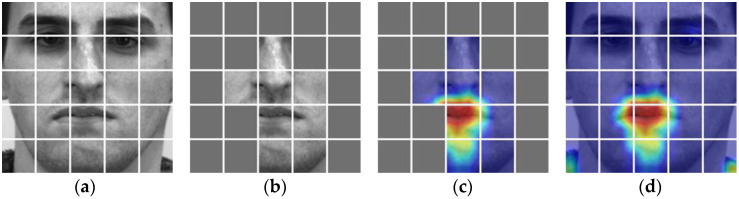
The correspondence between learnable class token and Grad-CAM in SAMM. (**a**) is a subblock of the original image; (**b**) is an image subblock that can learnable class token masks; (**c**) is the learnable class token mask image subblock corresponding to Grad-CAM; (**d**) is a Grad CAM image.

The performance and scale of the AUDF model are largely determined by learning mask marks. To further explain the impact of learning mask marks on model performance, the performance of AUDF is visualized through Grad-CAM. Figure 6, Figure 7 and Figure 8 can clearly show the corresponding relationship between learning mask marks and Grad-CAM in SMIC, CASME II, and SAMM datasets, where the first column is the sub-block division of the original image, and the second column is the visual representation of the mask of the original image by the LCT module, the third column is the mask representation of LCT in Grad-CAM, and the fourth column is the visual representation of Grad-CAM. It can be seen from the figure that LCT can mask out areas that have little influence on category weights and propose emotional features in local areas with high attention weights for micro-expression recognition.

## 5. Conclusions

In this paper, a multimodal dynamic attention fusion network method is proposed to enhance the local features of facial images by facial action unit embedding. To the parameter complexity of the vision transformer model, a learnable class token is proposed to sample a subset of patches with high attention weights to reduce the computational complexity of facial image feature extraction. The action unit dynamic fusion module is used to add action unit embedding information in the process of facial image local feature extraction to improve the distinguishability of image emotional features. The performance of the model is evaluated and verified on SMIC, CASME II, SAMM, and their combined 3DB-combined datasets. The experimental results show that the MADFN model can perform feature fusion through dynamic mapping, which can help improve the performance of micro-expression recognition.

The research related to micro-expression analysis in this paper mainly discusses the micro-expression recognition in determined videos, but often there is still how to locate the occurrence of micro-expressions in the real environment. In a real environment, the occurrence of micro-expressions is often to conceal one’s true emotions, so micro-expressions are often accompanied by the occurrence of macro-expressions. How to locate the location of micro-expressions from the complex environment and emotional changes is also key research in future work.

## Figures and Tables

**Table 1:** The performance comparison of MADFN and two types of models on the SMIC dataset.

Methods	Accuracy (%)	F1-Score
LBP-TOP (ICCV 2011)	48.78	0.4600
DiSTLBP-RIP (FADS 2019)	63.41	N/A
LBP-SDG (NC 2021)	69.68	0.6200
LBP-FIP (MTAP 2022)	67.86	N/A
KTGSL (NC 2022)	**75.64**	0.6900
OFF-Apex (SPIC 2019)	67.68	0.6709
DSSN (ICIP 2019)	63.41	0.6462
TSCNN (IEEE Access 2019)	72.74	**0.7236**
GEME (NC 2021)	64.63	0.6158
MoCo (PRL 2023)	75.61	0.7492
RPCA (ECCV 2014)	58.00	0.6000
MDMO (TAC 2015)	58.97	0.5845
FDM (TAC 2017)	54.88	0.5380
Bi-WOOF (SPIC 2018)	61.59	0.6110
FHOFO (TAC 2019)	51.83	0.5243
SMDMO (TAC 2021)	**70.51**	**0.7041**
CBAM (Information 2020)	54.84	N/A
RNMA (NC 2020)	49.40	0.4960
LGCconD (TIP 2020)	63.41	0.6200
MADFN	**81.71**	**0.8202**

**Table 2:** The performance comparison of MADFN and two types of models on the CAMSE II dataset.

Methods	Accuracy (%)	F1-Score
LBP-TOP (ICCV 2011)	39.68	0.3589
LTOGP (ICASSP 2019)	66.00	N/A
DiSTLBP-RIP (FADS 2019)	64.78	N/A
LBP-SDG (NC 2021)	71.32	0.6700
LBP-FIP (MTAP 2022)	70.00	N/A
KTGSL (NC 2022)	72.58	0.6800
OFF-Apex (SPIC 2019)	68.94	0.6967
DSSN (ICIP 2019)	70.78	0.7297
TSCNN (IEEE Access 2019)	80.97	0.8070
Graph-TCN (MM 2020)	73.98	0.7246
GEME (NC 2021)	64.63	0.6158
MoCo (PRL 2023)	76.30	0.7366
FDCN (SIVP 2023)	73.09	0.7200
MERSiamC3D (NC 2021)	**81.89**	**0.8300**
RPCA (ECCV 2014)	49.00	0.5100
MDMO (TAC 2015)	51.69	0.4966
FDM (TAC 2017)	45.93	0.4053
Bi-WOOF (SPIC 2018)	57.89	0.6125
FHOFO (TAC 2019)	56.64	0.5248
RAM (FG 2020)	68.20	0.5700
SMDMO (TAC 2021)	66.95	0.6911
CBAM (Information 2020)	69.92	N/A
RNMA (NC 2020)	65.90	0.5390
LGCconD (TIP 2020)	65.02	0.6400
AU-GCN (CVPR 2021)	74.27	0.7047
TSGACN (CVPR 2021)	**81.30**	**0.7090**
MADFN	**82.11**	**0.8095**

**Table 3:** The performance comparison of MADFN and two types of models on the SAMM dataset.

Methods	Accuracy (%)	F1-Score
LBP-TOP (ICCV 2011)	35.56	0.3589
KTGSL (NC 2022)	56.11	0.4900
DSSN (ICIP 2019)	57.35	0.4644
TSCNN (IEEE Access 2019)	71.76	0.6942
Graph-TCN (MM 2020)	75.00	0.6985
GEME (NC 2021)	64.63	0.6158
MoCo (PRL 2023)	68.38	0.7366
FDCN (SIVP 2023)	58.07	0.5700
MERSiamC3D (NC 2021)	68.75	0.5436
RNMA (NC 2020)	48.50	0.4020
LGCconD (TIP 2020)	40.90	0.3400
AU-GCN (CVPR 2021)	74.26	0.7045
TSGACN (CVPR 2021)	**88.24**	**0.8279**
MADFN	**77.21**	**0.7489**

**Table 4:** The performance comparison of MADFN and two types of models on the MEGC2019 dataset.

Methods	SMIC		CASME II		SAMM		3DB-Combined	
	UF1	UAR	UF1	UAR	UF1	UAR	UF1	UAR
LBP-TOP (ICCV 2011)	0.2000	0.5280	0.7026	0.7429	0.3954	0.4102	0.5882	0.5785
Bi-WOOF (SPIC 2018)	0.5727	0.5829	0.7805	0.8026	0.5211	0.5139	0.6296	0.6227
OFF-Apex (SPIC 2019)	0.6817	0.6695	0.8764	0.8681	0.5409	0.5409	0.7196	0.7096
CapsuleNet (FG 2019)	0.5820	0.5877	0.7068	0.7018	0.6209	0.5989	0.6520	0.6506
Dual-Inception (FG 2019)	0.6645	0.6726	0.8621	0.8560	0.5868	0.5663	0.7322	0.7278
STSTNet (FG 2019)	0.6801	0.7013	0.8382	0.8686	0.6588	0.6810	0.7353	0.7605
EMR (FG 2019)	**0.7461**	**0.7530**	0.8293	0.8209	**0.7754**	0.7152	**0.7885**	0.7824
SHCFNet (2020)	0.6100	0.6311	0.6540	0.6536	0.6089	0.5926	0.6242	0.6222
MERSiamC3D (NE 2021)	0.7356	0.7598	0.8818	0.8763	0.7475	**0.7280**	0.8068	**0.7986**
FeatRef (PR 2022)	0.7011	0.7083	**0.8915**	**0.8873**	0.7372	0.7155	0.7838	0.7832
GEME (NC 2021)	0.6288	0.6570	0.8401	0.8508	0.6868	0.6541	0.7395	0.7500
Bi-WOOF (SPIC 2018)	0.5727	0.5829	0.7805	0.8026	0.5211	0.5139	0.6296	0.6227
RCN-A (TIP 2020)	0.6441	0.6326	0.8123	0.8512	0.6715	0.7601	0.7190	0.7432
RCN-S (TIP 2020)	0.6572	0.6519	0.7914	0.8360	0.6565	0.7647	0.7106	0.7466
RCN-W (TIP 2020)	0.6600	0.6584	0.8131	0.8522	0.6164	0.7164	0.7100	0.7422
RCN-F (TIP 2020)	0.5980	0.5991	0.8087	0.8563	0.6771	0.6976	0.7052	0.7164
LGCcon (TIP 2021)	N/A	N/A	0.7929	0.7639	0.5248	0.4955	0.7914	0.7933
LGCconD (TIP 2020)	0.6195	0.6066	0.7762	0.7499	0.4924	0.4711	0.7715	0.7864
AU-GCN (CVPR 2020)	**0.7192**	0.7215	0.8798	0.8710	**0.7751**	**0.7890**	0.7979	0.8041
PLAN_S (NN 2022)	0.7127	**0.7256**	0.8632	0.8778	0.7164	0.7418	0.7826	0.7891
PLAN (NN 2022)	N/A	N/A	**0.8941**	**0.8962**	0.7358	0.7687	**0.8075**	**0.8013**
MADFN	**0.8179**	**0.8102**	**0.9061**	**0.8986**	**0.8322**	**0.8289**	**0.8100**	**0.8044**

**Table 5:** The different backbone model structure settings.

Model	Patch Size	Layers	Hidden Size	MLP Size	Heads
ViT-Base	16 × 16	12	768	3072	12
ViT-Large	16 × 16	24	1024	4086	16
ViT-Huge	14 × 14	32	1280	5120	16

**Table 6:** The influence of different backbone models.

Model	SMIC		CASME II		SAMM	
	Accuracy	F1-Score	Accuracy	F1-Score	Accuracy	F1-Score
ViT-B/16	64.63	0.6602	53.79	0.4780	58.19	0.3598
ViT-L/16	68.29	0.6858	57.24	0.5136	62.70	0.5280
ViT-H/14	65.24	0.6412	58.62	0.5386	59.69	0.4704

**Table 7:** The influence of different mask sampling strategies.

Model	SMIC		CASME II		SAMM	
	Accuracy	F1-Score	Accuracy	F1-Score	Accuracy	F1-Score
BS	65.85	0.6658	48.17	0.5069	68.38	0.6643
GS	65.24	0.6696	48.17	0.5008	67.64	0.6481
RS	68.29	0.6822	59.79	0.6033	69.11	0.6819
LS	69.51	0.7008	72.63	0.7327	72.46	0.7082

**Table 8:** The influence of different fusion strategies.

Methods	Image	AU	SMIC		CASME II		SAMM	
			Accuracy	F1-Score	Accuracy	F1-Score	Accuracy	F1-Score
ViT	√		69.51	0.7008	72.63	0.7327	72.46	0.7082
ViT-C-AU	√	√	71.85	0.7026	73.17	0.7327	73.44	0.7181
ViT-S-AU	√	√	71.68	0.7030	73.62	0.7386	73.70	0.7180
ViT-M-AU	√	√	73.51	0.7208	74.63	0.7427	74.11	0.7219
AUDF	√	√	78.04	0.7784	77.64	0.7520	75.73	0.7316
AUDF-E	√	√	**81.71**	**0.8202**	**82.11**	**0.8095**	**77.21**	**0.7489**

**Table 9:** The total number of parameters and training and testing time.

Model	Params	Training Times	Test Times
ViT-Base	86M	7.3H	13MS
ViT-Large	307M	10.6H	18MS
ViT-Huge	632M	24.2H	24MS
MADFN	224M	9.2H	25MS

## Data Availability

Publicly available SMIC, CASME II, and SAMM datasets were analyzed in this study. The SMIC dataset can be found here: https://www.oulu.fi/cmvs/node/41319 (accessed on 11 September 2018). The CASME II dataset can be found here: http://fu.psych.ac.cn/CASME/casme2.php (accessed on 11 September 2018). The SAMM dataset can be found here: http://www2.docm.mmu.ac.uk/STAFF/M.Yap/dataset.php (accessed on 16 September 2018).
